# Dried blood spot quality in newborn inherited metabolic disease screening in Beijing, 2020–2024: a retrospective analysis of incidence, causes, and improvement strategies

**DOI:** 10.1186/s12887-026-07116-7

**Published:** 2026-06-09

**Authors:** Lijin Gu, Jinqi Zhao, Lifei Gong, Yue Tang, Lulu Li, Shunan Wang, Wan Yang, Yuanyuan Kong

**Affiliations:** Department of Newborn Screening Center, Beijing Obstetrics and Gynecology Hospital, Capital Medical University/Beijing Maternal and Child Health Care Hospital, Beijing, 100026 China

**Keywords:** Newborn screening, Inherited metabolic diseases, Unqualified dried blood spot, Quality control, Retrospective study

## Abstract

**Background:**

This study focuses on assessing the quality of dried blood spots (DBS) for newborn inherited metabolic disease screening in Beijing from 2020 to 2024, identify the causes and risk factors of unqualified DBS, and provide evidence-based quality improvement strategies for clinical practice and public health management.

**Methods:**

We retrospectively analyzed all DBS samples collected from all obstetric institutions in Beijing. Data were extracted from the Beijing Newborn Disease Screening Management Information System. Unqualified DBS rates were analyzed by year, season, hospital type, and annual screening volume using chi-square tests, chi-square trend tests, and Bonferroni correction.

**Results:**

A total of 714,369 DBS samples were included in the study, of which 1,735 (0.24%) were unqualified. The recall rate for unqualified samples was 98.96%. The unqualified rate declined significantly from 0.33% in 2021 to 0.17% in 2024 (*P* < 0.001). The leading causes were incorrect/incomplete information filling (30.95%), unclear QR codes (18.44%), and blood seepage rings from repeated dipping (15.33%). The unqualified rate was highest in winter (0.31%, *P* < 0.001). Non-maternal and child health specialty hospitals had a higher rate than maternal and child health specialty hospitals (0.27% vs. 0.20%, *P* < 0.001). Institutions with ≤ 1,500 annual screening cases had a markedly higher rate than those with > 1,500 cases (0.40% vs. 0.16%, *P* < 0.001).

**Conclusions:**

DBS quality in Beijing is at an advanced international level, with a low unqualified rate and high recall rate. However, information-related errors and technical collection problems remain prominent. Targeted strategies include optimizing the information management system, implementing hierarchical training, strengthening three-level quality control, and establishing seasonal early warning mechanisms, particularly for non-maternal and child health hospitals and low-volume institutions.

## Introduction

Newborn screening for inherited metabolic diseases is a population-based secondary preventive measure for the early detection and initiation of therapy for all newborns with treatable endocrine and metabolic diseases. Left untreated, these diseases may lead to severe disabilities or even death [1]. It is one of the most cost-effective public health interventions in pediatric care. Dried blood spot (DBS) collection is a key step in this screening process. The quality of DBS plays a crucial role in the entire newborn disease screening process. Poor DBS quality can directly affect the accuracy of laboratory test results, potentially leading to false-negative outcomes that may cause affected infants to miss the optimal treatment window, thereby compromising their health [2, 3]. Unqualified DBS may delay analysis and increase turnaround time, raising healthcare costs [4, 5]. Additionally, unnecessary repeat sampling and false-positive results caused by unqualified DBS can provoke anxiety among parents of newborns, ultimately undermining the overall efficiency and credibility of the newborn screening system [6–10]. These findings collectively demonstrate that DBS quality serves as a critical prerequisite and core quality control checkpoint for ensuring the effectiveness of the entire newborn genetic metabolic disease screening process.

Internationally, studies from developed countries have extensively documented quality control practices in newborn DBS screening, with a strong focus on pre-analytical errors, specimen collection techniques, and short-term program performance. However, large-sample, long-term trend analyses examining the dynamic changes in unqualified DBS rates remain rare globally [11–14].

In China, regional studies have explored DBS quality issues in newborn screening, but most focus on single provinces or short-term data, with limited large-sample, long-term analyses of urban screening systems with mature quality control mechanisms [15–17]. Beijing has established a municipal-district-institutional three-level newborn screening management system, and its DBS quality has improved steadily in recent years. However, the specific long-term trends of unqualified DBS, their key influencing factors, and targeted, implementable quality improvement strategies remain to be systematically elucidated.

This study conducted a retrospective analysis of all DBS samples collected from all obstetric institutions in Beijing over a 5-year period (2020–2024).

### Primary objective

To determine the overall incidence and annual trends of unqualified dried blood spot (DBS) samples collected from all obstetric institutions across Beijing over a 5-year period (2020–2024), and to identify the primary causes of unqualified DBS samples and characterize their relative contributions to overall sample inadequacy.

### Secondary objectives

1. To evaluate the association between unqualified DBS rates and potential risk factors, including collection season, hospital type, and institutional screening volume.
2. To compare inter-institutional and inter-annual variation in DBS sample quality across participating obstetric facilities.
3. To develop evidence-based quality improvement strategies targeting the most prevalent and modifiable causes of unqualified DBS samples.
4. To provide a generalizable reference framework for optimizing newborn screening programs in China and in other countries or regions currently establishing or refining their screening systems.

## Methods

### Study subjects and design

This was a retrospective study involving all DBS samples used for newborn genetic metabolic disease screening from all obstetric institutions in Beijing between January 1, 2020, and December 31, 2024. All specimens were primary blood collections from newborns, with the entire process of DBS collection and submission conducted in strict accordance with the *Technical Specifications for Newborn Disease Screening (2010 Edition)* issued by the former Ministry of Health of China. All study data were retrieved from the Beijing Newborn Disease Screening Management Information System.

In China, newborn screening for inherited metabolic diseases is a legally mandated public health program. In Beijing, it is offered free of charge to all newborns, with the cost covered by the municipal government. Before sample collection, parents or legal guardians receive standardized verbal and written information about the purpose, procedure, benefits, and potential risks of screening, and written informed consent is obtained. The Beijing screening panel currently includes 12 inherited metabolic diseases (e.g., phenylketonuria, congenital hypothyroidism, congenital adrenal hyperplasia, and others). Positive cases are notified by dedicated staff of the Beijing Newborn Screening Center via telephone to arrange repeat blood sampling for confirmatory testing. If a positive case does not attend the follow‑up visit within one week, the relevant information is returned to the district‑level maternal and child health institution for continued tracking.

According to national regulations, the National Health Commission of China oversees the national newborn screening program, and provincial-level maternal and child health hospitals serve as regional screening centers. As the only newborn screening center in Beijing, the Beijing Maternal and Child Health Hospital undertakes centralized testing and management of all free newborn screening in the city. Through centralized procurement, it provides standard blood collection needles, filter paper cards, and related consumables to all obstetric institutions. A unique two-dimensional QR code is generated for each newborn per screening event. The screening record of each newborn is linked to live birth records in the Beijing Maternal and Child Health Information System, ensuring coverage of nearly all live births. During the study period, the screening coverage rate in Beijing remained above 98%.

Heel blood samples were collected after the newborn had been adequately breastfed, typically between 72 h and 7 days after birth (extended to a maximum of 20 days under special clinical circumstances), and applied onto dedicated filter paper. The collection was performed by trained nurses from obstetric institutions in the neonatal unit or postpartum ward of the hospital (or in the obstetrics outpatient clinic for those requiring post-discharge collection). The blood spots were air-dried naturally, sealed in sterile bags, and stored at 2–8 °C. They were then transported by dedicated couriers using cold-chain logistics to district-level maternal and child health institutions for review and verification. In the rare cases of home births (accounting for < 0.1% of live births in Beijing) [18], the district-level maternal and child health institution was responsible for arranging DBS collection within 7 days after birth. All samples were then shipped via cold-chain from the district-level institutions to the Beijing Newborn Screening Center for centralized and unified testing.

### Acceptability criteria for DBS

A DBS was considered qualified if it met all of the following criteria: (1) collected 72 h to 7 days after birth, with the newborn having received sufficient breastfeeding; (2) contained at least three independent blood spots, each with a minimum diameter of 8 mm; (3) showed natural penetration of blood drops through the filter paper, with consistent morphology on both sides; (4) had no contamination, blood seepage, or hemolysis; and (5) had complete and accurate documentation, including screening identification number, collector’s name, institution, maternal name, parity, gestational age, hospital admission number, two-dimensional QR code, newborn birth weight, sex, household registration, date of birth, collection date, parental contact information, and signed informed consent. Any DBS failing to meet one or more of these criteria was defined as unqualified. The criteria apply to all newborns regardless of feeding type, gestational age or birth weight, including preterm infants (< 37 weeks) and term infants (≥ 37 weeks).

### Grouping criteria

#### Seasonal grouping

Samples were categorized by blood collection date into four seasons: spring (March, April, May), summer (June, July, August), autumn (September, October, November), and winter (December, January, February).

#### Hospital type grouping

Collection institutions were divided into maternal and child health specialty hospitals and non-maternal and child health specialty hospitals based on their institutional nature.

#### Annual screening volume grouping

Institutions were stratified by their annual volume of newborn screening DBS collections into two groups: those with ≤ 1500 cases per year and those with > 1500 cases per year.

### Statistical analysis

IBM SPSS Statistics 25.0 software was used for all statistical analyses of the study data. Count data were expressed as numbers (*n*) and percentages (%). The chi-square test was applied to compare differences in the unqualified DBS rate among different groups. The chi-square trend test was used to analyze the annual variation trend of the unqualified DBS rate during the study period. Bonferroni correction was adopted for pairwise comparisons of the unqualified rate across the four seasonal groups, with statistical significance set at *P* < 0.0083 after Bonferroni correction. A two-tailed *P*-value < 0.05 was deemed statistically significant for all other statistical comparisons.

### Key quality improvement measures of the newborn screening system in Beijing

**Fig. 1:**
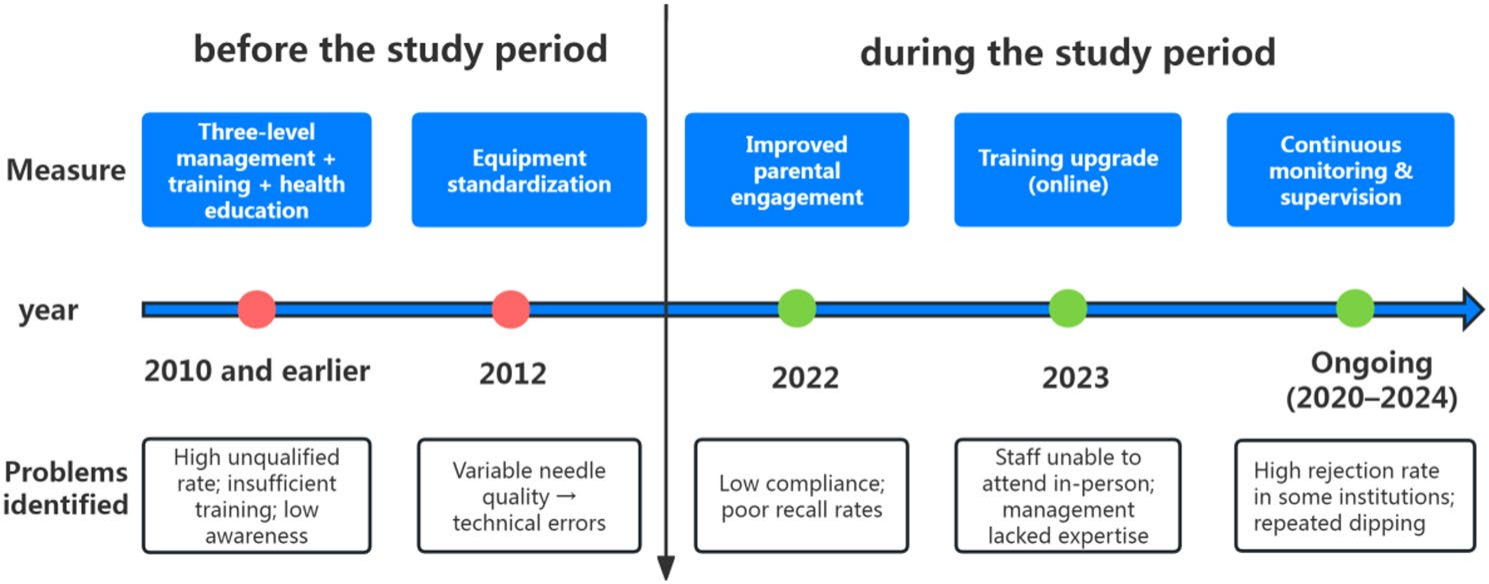
Timeline of key problems and solutions for the newborn screening system in Beijing

To provide context for the observed decline in unqualified DBS rates during the study period (2020–2024), we summarized the major quality improvement measures implemented in Beijing’s newborn screening system before and during the study period (Fig. 1). For each time period, the figure indicates the problems identified and the solutions introduced, including the establishment of the three‑level management and training system (2010 and earlier), equipment standardization (2012), improved parental engagement (2022), training upgrade (2023), and continuous monitoring and supervision (ongoing 2020–2024). This timeline provides context for the declining unqualified DBS rates observed in the Results.

## Results

### General incidence and recall of unqualified DBS

During the study period (2020–2024), there were 715,815 live births in Beijing. Of these, 714,369 newborns (≥ 99% coverage) were screened for inherited metabolic diseases, with the screening coverage rate remaining above 98% throughout the period (as noted in the Methods section). Among the screened newborns, 1,735 DBS samples were identified as unqualified, yielding an overall unqualified rate of 0.24%. A total of 1,717 unqualified samples were successfully recalled, corresponding to a recall rate of 98.96%. The chi-square trend test showed that the rate of unqualified DBS had a significant downward trend year by year (χ²=100.203, *P* < 0.001), with the highest rate in 2021 (0.33%) and the lowest in 2024 (0.17%). The recall rate of unqualified DBS was above 98% in each year, with the highest rate in 2024 (99.59%) (Table 1).

**Table 1:** Incidence and recall of unqualified DBS in newborn inherited metabolic disease screening in Beijing, 2020–2024

Year	Screened (*n*)	unqualified (*n*)	Unqualified rate (%)	Recalled (*n*)	Recalled rate (%)
2020	161,508	439	0.27	434	98.86
2021	146,615	481	0.33	474	98.54
2022	132,697	332	0.25	328	98.80
2023	128,994	239	0.19	238	99.58
2024	144,555	244	0.17	243	99.59
Total	714,369	1735	0.24	1717	98.96

### Causes of unqualified DBS

Information-related problems were the primary cause of unqualified DBS, accounting for 57.75% (1,002/1,735), followed by blood spot collection technical problems (37.41%, 649/1,735) and other reasons (4.84%, 84/1,735). Information-related problems were the most common cause in each year during the study period.

**Fig. 2:**
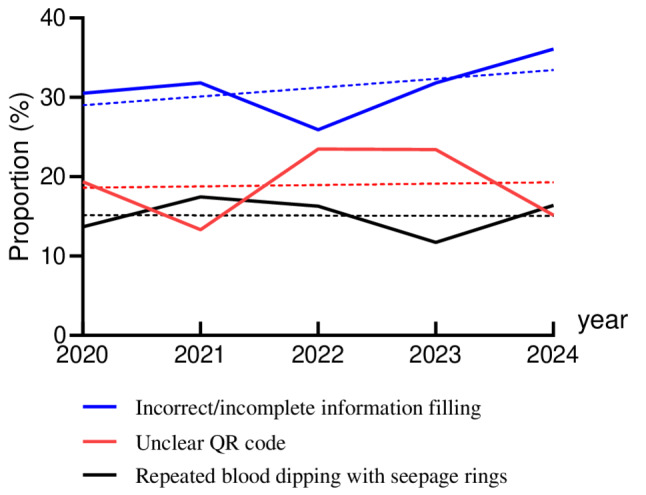
Trends in the top three causes of unqualified DBS in Beijing, 2020–2024

In terms of specific causes, the top three were incorrect/incomplete information filling (30.95%, 537/1,735), unclear QR codes that could not be scanned into the information system (18.44%, 320/1,735), and blood seepage rings caused by repeated blood dipping (15.33%, 266/1,735) (Table 2). Trend analysis of the main causes showed that the proportion of information entry errors/incompleteness increased from 30.52% in 2020 to 36.07% in 2024, but the difference was not statistically significant (*χ*² = 7.228, *P* > 0.05).The proportion of blood seepage rings caused by repeated blood soaking increased from 13.46% in 2020 to 16.39% in 2024, but the difference was not statistically significant (*χ*² = 5.463, *P* > 0.05).In contrast, the proportion of unclear QR code information decreased from 19.36% in 2020 to 15.16% in 2024, and the difference was statistically significant (*χ*² = 20.014, *P* < 0.001) (Fig. 2).

**Table 2:** Causes of unqualified DBS in newborn inherited metabolic disease screening in Beijing, 2020–2024

Category Specific cause		*n*	Constituent ratio (%)
Blood spot collection technical problems	Repeated blood dipping with seepage rings	266	15.33%
	Blood spot contamination	132	7.61%
	Uneven penetration	103	5.94%
	Incomplete penetration on both sides	80	4.61%
	Small blood spots	42	2.42%
	Blood dilution	20	1.15%
	Tissue fluid exudation	6	0.35%
	Subtotal	649	37.41%
Information-related problems	Incorrect/incomplete information filling	537	30.95%
	Unclear QR code (unscannable)	320	18.44%
	Signature not captured/incorrect signature of parents/collectors	145	8.36%
	Subtotal	1002	57.75%
Other reasons	Expired filter paper	43	2.48%
	Only 2 blood spots	25	1.44%
	Misuse of deafness gene card (no discard of first blood drop)	8	0.46%
	Cut/ torn blood spots	6	0.35%
	Blood collection within 72 h after birth	2	0.12%
	Subtotal	84	4.84%
Total		1735	100.00%

### Factors associated with unqualified DBS

#### Seasonal difference

There were statistically significant differences in the unqualified rate among different seasons (*χ*² = 40.867, *P* < 0.001). Pairwise comparison showed that the unqualified rate in winter (0.31%, 537/174,840) was significantly higher than that in spring (0.22%, 392/179,239), summer (0.21%, 389/181,357) and autumn (0.23%, 417/178,933) (all *P* < 0.0083). No statistically significant differences were found in the unqualified rate among spring, summer and autumn (all *P* > 0.0083).

#### Difference by hospital type

The unqualified rate in non-maternal and child health specialty hospitals (0.27%, 1259/472,200) was significantly higher than that in maternal and child health specialty hospitals (0.20%, 476/242,169), with a statistically significant difference (*χ*² = 32.148, *P* < 0.001).

#### Difference by annual screening volume

The unqualified rate in institutions with an annual screening volume ≤ 1500 cases (0.40%, 949/235,676) was significantly higher than that in institutions with > 1500 cases (0.16%, 786/478,693), with a statistically significant difference (*χ*² = 369.708, *P* < 0.001) (Table 3).

Further analysis of non-maternal and child health specialty hospitals showed that the unqualified rate in institutions with a screening volume ≤ 1500 cases (0.36%, 632/175,660) was significantly higher than that in institutions with > 1500 cases (0.21%, 627/296,540), with a statistically significant difference (*χ*² = 91.296, *P* < 0.001) (Table 4).

**Table 3:** Comparison of intra-group differences in the incidence of unqualified DBS in Beijing, 2020–2024

Classification	Number	Unqualified rate [*n*(%)]	χ²	*P* value
Collection season			40.867	< 0.001
Spring	179,239	392 (0.22%)		
Summer	181,357	389 (0.21%)		
Autumn	178,933	417 (0.23%)		
Winter	174,840	537 (0.31%)		
Hospital type			32.148	< 0.001
Maternal and child health specialty hospitals (32 hospitals)	242,169	476 (0.20%)		
Non-maternal and child health specialty hospitals (84 hospitals)	472,200	1259 (0.27%)		
Annual screening volume			369.708	< 0.001
≤ 1500 cases (85 hospitals)	235,676	949 (0.40%)		
> 1500 cases (31 hospitals)	478,693	786 (0.16%)		

The“Number” column refers to the total number of DBS samples (i.e., newborns screened) collected over the five-year period. Due to the annual opening or closure of some institutions and variations in screening volume, the number of hospitals in each category is presented as the five-year average

**Table 4:** Comparison of the incidence of unqualified DBS by screening volume in non-maternal and child health specialty hospitals in Beijing, 2020–2024

Annual screening volume	Number	Unqualified rate [*n*(%)]	χ²	*P* value
≤ 1500 cases (60 hospitals)	175,660	632 (0.36%)	91.296	< 0.001
> 1500 cases (24 hospitals)	296,540	627 (0.21%)		

The“Number” column refers to the total number of DBS samples (i.e., newborns screened) collected over the five-year period. Due to the annual opening or closure of some institutions, the number of hospitals in the category is presented as the five-year average

## Discussion

### Overall DBS quality in Beijing is at an advanced international level

From 2020 to 2024, the overall rate of unqualified dried blood spot (DBS) samples in newborn screening for inherited metabolic diseases in Beijing was 0.24%, which was substantially lower than the rates reported in other regions of China, including Inner Mongolia (4.96%) [15], Gansu (1.27%) [16], and Guangxi (0.40%) [17]. Moreover, this rate not only met but surpassed the internationally recommended benchmark, as the United Kingdom defines an acceptable avoidable re-collection rate as ≤ 1% [11]. It was also lower than the rates reported in the Philippines (3.51%) [19] and Romania (approximately 1%) [20], and was comparable to the rejection rate reported in Saudi Arabia (0.51%) [5].

The year-by-year decline in the unqualified DBS rate from 0.33% (2021) to 0.17% (2024) reflects the sustained impact of quality improvement measures implemented in Beijing before and during the study period. A detailed timeline of these measures, including their start years, is provided in the Methods section ( Fig. 1). Key actionable steps derived from this experience for other institutions include: (1) standardizing collection equipment through unified procurement; (2) implementing mandatory pre-job certification and ongoing online training for collection personnel (also covering district-level management staff); (3) establishing a three‑tier quality control system with regular targeted on-site and online supervision; and (4) expanding parental health education using standardized prenatal class materials.These measures align with internationally recognized quality improvement principles that emphasize standardized training and quality feedback [21].

The recommended sampling window in Beijing (72 h to 7 days) is longer than the typical 48–72 h window used in Germany [22] and Norway [23] and much longer than the 24–48 h window common in many US states [24, 25]. This extended window was adopted to reduce false positives by allowing metabolic stabilization (e.g., avoiding the physiological TSH surge before 72 h). Our data show that only 0.12% of unqualified samples (*n* = 2, Table 2) were collected before 72 h, indicating excellent adherence to the lower limit. However, later sampling may delay diagnosis. Balancing timeliness and accuracy remains a consideration for future guideline updates.

### Information-related problems remain the primary bottleneck

Information-related problems accounted for more than half of the causes of unqualified DBS, and the incorrect/incomplete information filling has become the most prominent quality problem at present. This type of problem is mainly related to the professional proficiency and sense of responsibility of blood collection and information entry personnel, and is more challenging to solve than hardware equipment problems (e.g., unclear QR codes). Incorrect or incomplete filling of key information (e.g., newborn weight, gestational age, contact phone number) not only affects the accurate interpretation of laboratory test results (the cut-off value of some metabolic disease indicators is related to weight and gestational age [3]) but also leads to failure in the follow-up of suspected positive cases and even loss of follow-up [26].

Although Beijing has applied a newborn screening information management system based on Hanxin codes since 2009 [27], the lack of logical verification functions in the system is an important reason for the high incidence of information errors, providing a feasible solution for future optimization. In addition, unclear QR codes — the second major information-related problem—are mainly due to the aging and inadequate maintenance of unified printers; although their proportion is declining, regular equipment maintenance and updates are still necessary. The third major information-related problem is the lack of signature or incorrect signature, which reflects the weak awareness of responsibility traceability among front-line staff and needs to be strengthened in daily management.

### Blood spot collection technical problems are prominent

Blood spot collection technical problems accounted for 37.41% of unqualified DBS, with repeated blood dipping leading to seepage rings as the most common problem (15.33%). Repeated blood dipping causes uneven chromatography of blood spots and serum extrusion, which seriously affects the elution efficiency of metabolites in the blood and the accuracy of test results, and may even lead to false negative results [6, 28]. Other technical problems such as blood spot contamination, uneven penetration, and incomplete penetration are mainly caused by non-standard operations of blood collection personnel, such as weak aseptic concepts and incorrect blood collection and squeezing techniques [29, 30].

The frequent occurrence of blood collection technique problems indicates that that training for front-line staff needs to be more targeted, especially practical operation training for key links such as standardized blood dipping and squeezing, and on-site supervision and error correction need to be strengthened. In addition, the simultaneous implementation of newborn inherited metabolic disease screening and deafness gene screening in Beijing has brought new quality risks (e.g., misuse of screening cards), and it is necessary to take targeted measures such as color-coding of cards and eye-catching operation flow charts to reduce operational errors.

### Seasonal, institutional, and volume-related factors identify priority targets

The unqualified rate in winter was significantly higher than that in other seasons, which is mainly related to the Spring Festival holiday and cold weather. During the holiday, regular nurses are often on rotation or leave, and temporary or less experienced staff may be assigned to blood collection duties. Cold weather easily causes insufficient blood volume due to inadequate hot compress of the blood collection site, thus increasing the incidence of technical problems such as repeated blood dipping [31]. Therefore, winter—especially around the Spring Festival—should be regarded as a key early warning period for DBS quality control, with enhanced pre-holiday training, reasonable staffing, and increased sampling frequency.

The unqualified rate in non-maternal and child health specialty hospitals was higher than that in maternal and child health specialty hospitals, and the rate in low-screening-volume institutions (≤ 1500 cases) was significantly higher than that in high-screening-volume institutions, which is consistent with the results of previous studies [31, 32]. The main reasons are that maternal and child health specialty hospitals (such as district-level maternal and child health institutions) are deeply involved in the daily management of newborn screening. Their leadership places great emphasis on newborn screening, treating it as a core service and establishing robust internal quality control systems. In contrast, in non-maternal and child health specialty hospitals, newborn screening is often just one of many tasks in obstetrics and is easily marginalized. These hospitals pay less attention to newborn screening and provide professional training less frequently.

The blood collection personnel in low-screening-volume institutions have low operational proficiency due to insufficient practice. Further analysis found that the unqualified rate of non-maternal and child health specialty hospitals with low screening volumes was higher, making this subgroup the key target for subsequent quality improvement. For these institutions, it is necessary to carry out small-class practical operation training, strengthen the double-check system for information and blood spot quality, and give full play to the hub role of district-level maternal and child health institutions in daily on-site guidance and supervision.

This study has several strengths: it is the first large-sample retrospective study on DBS quality in newborn screening in northern China, with the unqualified rate reaching the international ideal level, and the findings can provide a reference for newborn screening quality control in other countries and regions.

This study has several limitations. First, as a retrospective analysis, it describes causes and influencing factors of unqualified DBS but does not track the specific impact of these quality defects on laboratory test indicators (e.g., false-negative/false-positive rates), clinical diagnosis delay or long-term prognosis of affected infants. Second, we did not collect data on individual collector characteristics (e.g., years of experience, training history), which may have provided additional insights. Third, the findings are from a single city with a well-established screening system, which may limit generalizability to regions with different healthcare structures. Fourth, detailed information on infant feeding type (e.g., exclusive breastfeeding, formula feeding, total parenteral nutrition) was not systematically captured in the screening database; therefore, we could not analyze its potential impact on DBS quality. Future studies should incorporate feeding data to assess this relationship and combine clinical follow-up data to evaluate the clinical risks and health economic losses associated with DBS quality problems.

At the international level, beyond traditional training and quality control, several recent technological advances offer promising avenues for further improving DBS quality. For example, researchers have developed a computer vision algorithm that can objectively measure DBS diameter and identify incorrectly applied blood spots, effectively reducing the subjectivity inherent in visual assessment [33]. Barcode-based tracking systems have been implemented to ensure accurate identification of infants and samples throughout the screening process, eliminating data transcription errors [34]. The updated 2025 General Guidelines of the International Society for Neonatal Screening provide a comprehensive framework for building and maintaining robust newborn screening systems [35]. These international experiences suggest that, in our setting, future improvements could include integrating automated image analysis into the quality control workflow, adopting digital tracking systems for sample traceability, and aligning local protocols with international guidelines to further reduce the unqualified rate.

## Conclusions

The quality of DBS for newborn inherited metabolic disease screening in Beijing has improved continuously, with a high recall rate of unqualified DBS and an overall unqualified rate at an advanced international level. However, information-related problems remain the primary cause of unqualified DBS, and collection technical problems such as repeated blood dipping are prominent and show an increasing trend. The unqualified rate is relatively high in non-maternal and child health specialty hospitals, low-volume screening institutions, and during winter.

To further improve DBS quality, we recommend the following strategies: (1) Optimize the newborn screening information management system and embed logical verification functions for key information to reduce information entry errors; (2) Conduct hierarchical and targeted training, with emphasize practical operation training for low-screening-volume institutions and non-maternal and child health specialty hospitals, and strengthen training on responsibility awareness; (3) Deepen the municipal-district-institutional three-level joint quality control mechanism, give full play to the on-site guidance role of district-level maternal and child health institutions, and implement differentiated supervision for key institutions; (4) Establish an early warning mechanism for key periods such as winter, strengthen pre-holiday quality control reminders and post-holiday sampling inspections, and ensure the standardization of blood collection work during holidays.

## Data Availability

The datasets generated and analyzed during the current study are not publicly available due to institutional data protection policies but are available from the corresponding author on reasonable request.

## Abbreviation

DBS: dried blood spots
